# The function of non-coding RNAs in idiopathic pulmonary fibrosis

**DOI:** 10.1515/med-2021-0231

**Published:** 2021-03-26

**Authors:** Hui Zhang, Miao Song, Jianing Guo, Junbing Ma, Min Qiu, Zheng Yang

**Affiliations:** Department of Cardiovascular Diseases, First Affiliated Hospital of Baotou Medical College, Baotou, Inner Mongolia, China; Department of Pharmacy, Baotou Medical College, Baotou, Inner Mongolia, China; Comfort Medical Center, Central hospital of Ulanqab, Ulanqab, Inner Mongolia, China

**Keywords:** ncRNAs, miRNAs, lncRNAs, IPF, pathogenesis

## Abstract

Non-coding ribonucleic acids (ncRNAs) are a diverse group of RNA molecules that are mostly not translated into proteins after transcription, including long non-coding RNAs (lncRNAs) with longer than 200 nucleotides non-coding transcripts and microRNAs (miRNAs) which are only 18–22 nucleotides. As families of evolutionarily conserved ncRNAs, lncRNAs activate and repress genes via a variety of mechanisms at both transcriptional and translational levels, whereas miRNAs regulate protein-coding gene expression mainly through mRNA silencing. ncRNAs are widely involved in biological functions, such as proliferation, differentiation, migration, angiogenesis, and apoptosis. Idiopathic pulmonary fibrosis (IPF) is a progressive lung disease with a poor prognosis. The etiology of IPF is still unclear. Increasing evidence shows the close correlations between the development of IPF and aberrant expressions of ncRNAs than thought previously. In this study, we provide an overview of ncRNAs participated in pathobiology of IPF, seeking the early diagnosis biomarker and aiming for potential therapeutic applications for IPF.

## 1 Introduction

Idiopathic pulmonary fibrosis (IPF) is a chronic, progressive type of interstitial pneumonia and is currently incurable. IPF is characterized by progressive lung scarring with constantly decreasing the function of the lungs. The prevalence of IPF shows that almost 5 million individuals suffer from this disease worldwide in the United States, and the 3 years and 5 years mortality rates of IPF are at approximately 50 and 80%, respectively [1]. Therefore, there is an urgent unmet need for deep understanding of the IPF biology, with a view to creating new possibilities of more effective targeted therapies. In the past decades, increasing studies reported that non-coding ribonucleic acids (ncRNAs) played important roles both in basic understanding of the molecular pathogenesis of IPF and for their use in novel therapeutic approaches.

ncRNAs, including microRNAs (miRNAs) and long non-coding RNAs (lncRNAs), are transcribed from human genome such as message RNAs (mRNAs) but are not translated into proteins after transcription. These ncRNAs directly or indirectly participate in the regulation of multiple functions of intracellular pathobiological processes and affect gene expression by regulating the function of mRNA, DNA methylation, and histone modification and participate in the occurrence and development of lung diseases, thus providing new ideas for the diagnosis and drug treatment targets of diseases [2,3,4].

## 2 Methods

**Figure 1:**
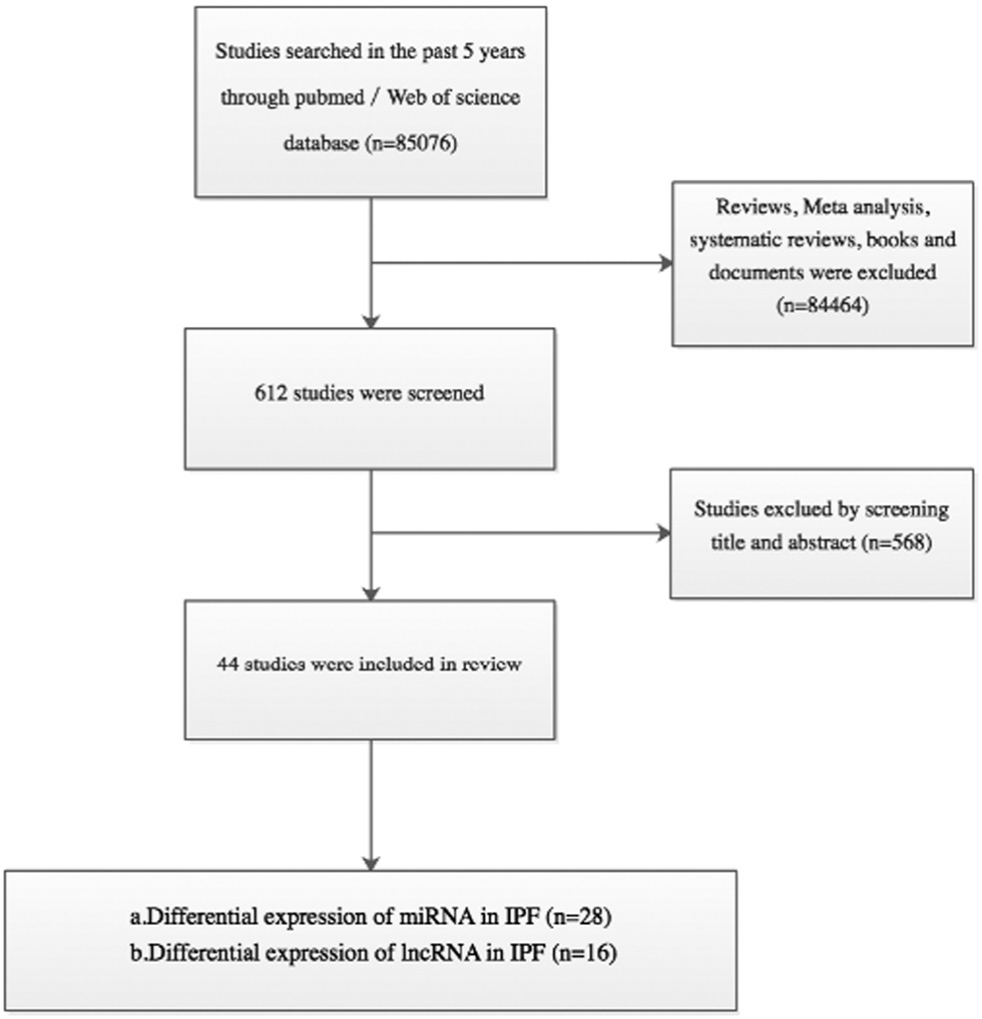
Flow chart for study selection.

The non-coding RNAs (ncRNAs), microRNAs (miRNAs), long non-coding RNAs (lncRNAs), idiopathic pulmonary fibrosis (IPF) were used as keywords searched in PubMed and web of science databases. The papers about reviews, meta-analysis, systematic reviews, books, and documents were excluded. Then, about 85,076 articles from PubMed and web of science databases in the past 5 years (2015–2019) (Figure 1) related to ncRNAs and IPF were screened and reviewed.

## 3 Result

The articles of ncRNAs and IPF are related in the following three areas: (1) the biogenesis and functions of miRNAs in IPF; (2) the biogenesis and functions of lncRNAs in IPF; and (3) the interaction networks among lncRNAs, miRNAs, and mRNA in IPF.

### 3.1 Expression changes and functions of miRNAs in IPF

#### 3.1.1 Biogenesis and biological functions of miRNAs

miRNAs are short single-stranded non-coding RNA molecules found in eukaryotic cells with a length of about 22nt, whose biological processes involve a series of complex steps. The precursor transcripts (pri-miRNAs) are a capped and polyadenylation transcript produced by RNA polymerase II [5]. Then, under the action of enzymes such as RNase III endonuclease and Drosha, the pri-miRNAs form precursor miRNAs (pre-miRNAs) with stem-looped structures and transport from the nucleus into the cytoplasm via the exportin-5 complex and the nuclear protein Ran-GTP [6,7]. In cytoplasm, the pre-miRNAs are further processed by Dicer into RNA duplex structure. Subsequently, the miRNA strand forms an RNA-induced silencing complex with the Argonaute protein and pairs with the corresponding target mRNA [8]. As the bases of the pairing sites are completely or incompletely complementary, the miRNA will degrade or block the translation of the mRNA.

miRNAs are involved in many biological processes such as biological development, cell proliferation and differentiation, tissue damage repair, and organ fibrosis and exert a wide range of physiological regulation [9,10,11]. In addition, a miRNA can affect the expression of multiple genes. In the meantime, a gene with multiple partial complementary binding sites can be targeted by multiple miRNAs [12]. Although several studies [13,14,15] have shown that there is a certain relationship between miRNA and the pathogenesis of IPF, regrettably, the specific mechanism remains unclear and needs further exploration.

#### 3.1.2 Diagnostic role of miRNAs in IPF

IPF is one of the most serious fibrotic pulmonary diseases, which commonly afflict the elderly. The main pathological manifestations are the alternation of fibrotic areas with mild or normal pulmonary parenchymal areas which simultaneously occur scar formation and honeycomb-like changes [16]. In short, the IPF is characterized by pulmonary interstitial fibrosis and respiratory function deterioration. miRNA has been found to be stably present in various body fluids such as blood, bronchoalveolar lavage fluid, and sputum and has the advantages of disease-specific expression and easy to detect and quantify [2]. It has become a good diagnostic biomarker for the pulmonary disease. Indeed, several studies have suggested that there are some significant differential expressions of miRNAs in the lung of IPF patients and play a vital role in the occurrence and development of IPF. Therefore, detecting the expression of these miRNAs in the body fluids of patients can predict IPF in advance or perform molecular targeted therapy to improve the prognosis of patients, which is a good biological indicator for the diagnosis and prognosis of IPF [17,18,19].

To explore the potential of miRNAs as biomarkers of IPF, Li et al. [17] analyzed the differential expression of serum miRNAs between IPF patients and healthy people by microarray analysis and biological functions analysis. Subsequently, the results were verified by quantitative reverse transcription-polymerase chain reaction (qRT-PCR). The biological function analysis results showed that there was a significant differential expression of 60 miRNAs in the serum of patients with IPF, including eight upregulated and 52 downregulated miRNAs. The gene ontology (GO) and Kyoto Encyclopedia of Genes and Genomes (KEGG) enrichment analysis of differential miRNAs expression pattern showed that the miRNAs might be involved in the biological process of IPF at different cellular levels. Furthermore, the qRT-PCR results combined with the microarray analysis results revealed that the expression level of miRNA-21, miRNA-155, and miRNA-101-3p in serum was significantly changed and were associated with decreased forced vital capacity (FVC) and lung injury levels in IPF patients. The above results indicate that miRNAs are involved in different biological processes in the development of IPF, and the expression level of miRNA can be used as an indicator for the diagnosis and prognosis of patients with IPF.

In a recent study, Njock et al. [18] found that three miRNAs were significantly abnormal in IPF patients by analyzing sputum exosome miRNA used miRNA quantitative PCR array. Further, they found a significant negative correlation between miR-142-3p and the ratio of pulmonary carbon monoxide diffusion volume to alveolar ventilation volume. This interesting study indicates that sputum exosomes miRNA may be a good marker for the diagnosis and pathological analysis of IPF.

Yang et al. [19] analyzed the human serum miRNAs among healthy subjects and rapid-progressing and slow-progressing IPF patients by TaqMan microRNA assay. The results showed that 47 signature miRNAs were expressed in IPF patients, including 21 upregulated and 26 downregulated miRNAs. Subsequently, these differentially expressed miRNAs were processed by bioinformatics analysis and verified six miRNAs (miR-21, miR-199a-5p, 32 miR-200c, miR-31, let-7a, and let-7d) by RT-PCR in three groups. KEGG signaling pathway enrichment analysis showed that differentially expressed serum miRNAs were significantly enriched in 53 signaling pathways, especially in TGF- β, MAPK, PI3K-Akt, and WNT signaling pathways, suggesting that these biological pathways were involved in the pathogenesis of IPF. Meanwhile, the results of RT-PCR suggested that patients with IPF had significantly higher expression of miR-21, miR-199a-5p, and miR-200c compared with healthy subjects, while miR-31, let-7a, and let-7d were significantly lower. The above results indicate the value of analyzing serum miRNAs to diagnose and monitor the prognosis of IPF.

#### 3.1.3 Therapeutic value of miRNA in IPF

IPF is because of the uncontrolled production of extracellular matrix by fibroblasts in lung injury, which leads to progressive exacerbations and irreversible damages [20]. Numerous studies [20,21,22,23] have shown that the abnormal expression of miRNA is closely related to the pathogenesis of IPF, therefore determining that the role of specific miRNA in the pathogenesis of IPF may provide a new direction for new therapeutic application. Ge et al. [22] used the bleomycin (BML)-mediated mouse lung fibrosis model to demonstrate the inhibited pulmonary fibrosis in mice by upregulating miR-323a-3p expression. Further studies have found that miR-323a-3p can target critical mediators in the pulmonary fibrosis pathways such as TGF-α, TGF-β, and apoptosis and attenuate these pathway signals, thereby reducing cell apoptosis. Therefore, miR-323a-3p can simultaneously target multiple fibrosis signaling pathways and can be further developed as an anti-IPF therapeutic agent.

At present, the mature IPF model is BML-induced pulmonary fibrosis model in mice. Zhang et al. [24] found that BML induced epithelial–mesenchymal transition (EMT), which promoted the migration of pleural mesothelial cells and subpleural pulmonary fibrosis. The downregulation of miR-18a-5p played a key role in the pathogenesis of pulmonary fibrosis. Furthermore, they found that miR-18a-5p binds to the transforming growth factor β receptor II (TGF-β-RII) mRNA, thereby reducing TGF-β-RII expression and downregulating the TGF-β/Smad2/3 signaling pathway. Then, they demonstrated that overexpression of miR-18a-5p reduced BML-induced EMT and reduced lung pleural fibrosis in mice. In addition, Liu et al. [25] demonstrated the important role of miR-21 in the pathogenesis of IPF. The miR-21 can regulate the expression of inhibitory Smad/Smad7 signaling pathway via TGF-β1, promote the activation of fibroblasts, and lead to pulmonary fibrosis. In summary, regulation of specific miRNA expression may be a new approach to the treatment of IPF.

The core pathogenesis of IPF is an abnormal epithelial–mesenchymal crosstalk, which hinders the normal repair process after alveolar damage [26,27,28]. Li et al. [29] also determined miRNA expression profiles in IPF patients and normal lungs by Affymetrix microarray. By analyzing the miRNA expression profiles by bioinformatics and pathway enrichment, they found that seven miRNAs were significantly reduced in the lung tissue of patients with IPF. Among them, miR-130b-3p was the most obvious reduction, and it is used as a research target miRNA. Subsequently, the target gene of miR-130b-3p was determined to be insulin-like growth factor (IGF-1) by fluorescein assay and ELISA reagent. To verify that miR-130b-3p and its target gene IGF-1 cause IPF through the disorder of epithelial–mesenchymal crosstalk, they first established a co-culture system of lung epithelial cells and fibroblasts to mimic the environment of epithelial–interstitial crosstalk. Then, they inhibited the expression of miR-130b-3p in epithelial cells, and the results showed that fibroblasts significantly increased the expression of collagen I, cell proliferation, and migration in the co-culture system. These fibrosis-promoting phenomena could be blocked by IGF-1 antibodies. Thus, the downregulation of miR-130b-3p leads to the secretion of IGF-1 by lung epithelial cells, which promotes the activation of fibroblast and the disorder of epithelial–interstitial crosstalk, and miR-130b-3p may play a key regulatory role in IPF therapy.

### 3.2 Expression changes and functions of lncRNAs in IPF

#### 3.2.1 Biogenesis and biological functions of lncRNAs

lncRNAs are ncRNAs greater than 200 nucleotides in length. lncRNAs lack coding open reading frames and cannot encode proteins, but they can exert biological functions through interaction with other genetic materials [30]. lncRNAs can be broadly classified into five categories according to the positional relationship with protein-coding genes, including sense lncRNAs, antisense lncRNAs, intronic lncRNAs, intergenic lncRNAs, and bidirectional lncRNAs [31]. Initially, lncRNA was considered to be a junk RNA because of its lack of biological function, only as a by-product of RNA polymerase II transcription and an interference factor for gene transcription. However, recent studies [32,33,34] have shown that lncRNAs have a variety of biological functions: (1) they participate in many important regulatory processes such as chromosome modification, transcriptional activation, and nuclear transport, (2) they protect protein-coding genes, and (3) they play a role in cis-regulation and trans-regulation. lncRNAs have multiple modes of action. For instance, lncRNAs can form complementary duplexes with transcripts-encoding protein genes to regulate gene expression levels [31]. Further, they can bind to specific proteins to form nucleic acid–protein complexes that regulate the protein activity or alter the protein localization in the cytoplasm [35,36].

Although lncRNAs are different from other nucleotide sequences and do not have high expression abundance, they are expressed at specific stages of biological development with high tissue-cell specificity and may become biomarkers of various diseases [37].

#### 3.2.2 Functions of lncRNAs in IPF

The above has shown that lncRNAs are involved in a variety of biological processes, and substantial evidence suggests that mutations and disorders of lncRNAs may be closely related to the pathogenesis of IPF.

Hao et al. [38] extracted venous blood RNA from IPF patients and healthy subjects for transcriptome sequencing and bioinformatics analysis. They found a total of 1,816 differentially expressed lncRNAs, including 440 upregulated lncRNAs and 1,376 downregulated lncRNAs. GO enrichment analysis and KEGG pathway enrichment analysis revealed that differential lncRNA was primarily involved in chromosome segregation and regulation and enriched in the mTOR signaling pathway associated with IPF. Studies have shown that the expression of mTOR pathway is associated with collagen production and pulmonary edema levels in IPF patients, while RPS6KB2 is an important gene in mTOR pathway. Therefore, RPS6KB2 can be activated by growth factors and regulate mTOR signaling pathway. Subsequently, RT-qPCR confirmed that lncRNA AP003419.16 and its adjacent gene RPS6KB2 were highly expressed in IPF patients but low expression in healthy people. TGF-β1 is a key pro-fibrotic factor in the pathogenesis of IPF. They found that AP003419.16 and RPS6KB were highly expressed in TGF-β1-treated A549 cells, which was consistent with the RT-qPCR results. Thus, the expression of lncRNA AP003419.16 can predict the risk of IPF and becomes a new target for IPF molecular therapy.

Song et al. [39] first discovered that lncRNA-ITPF (lncITPF) was upregulated in IPF. Various experimental techniques such as RNA fluorescence *in situ* hybridization, nuclear isolation, and RNA sequencing have confirmed that lncITPF is located in the nucleus and regulates its host gene ITGBL1 to promote fibrosis by affecting the acetylation of H3 and H4 histones in its promoter region. Then, they studied the upstream and downstream mechanisms of lncITPF in pulmonary fibrosis. The upstream mechanism of lncITPF is mediated by the TGF-β1/Smad2/3 signaling pathway, and the downstream mechanism regulates the acetylation of H3 and H4 histones of ITGBL1 gene by interacting with heterogeneous nuclear ribonucleoprotein L. To assess the potential of lncITPF as a therapeutic target, interfered sequence of lncITPF (sh-lncITPF) was applied to an animal model of pulmonary fibrosis, and the expression of lncITPF in clinical IPF patients was detected. The results showed that the fibrosis indexes of α-sma, collagen, and vimentin in the sh-lncITPF group were lower than those in the control group. The expression level of lncITPF in clinical IPF patients was significantly higher than that in healthy subjects, and the Pearson’s correlation analysis showed that lncITPF expression level was correlated with FVC% predicted. Therefore, lncITPF may become one of the new targets for clinical IPF treatment.

In addition, Gao et al. [40] first discovered that lncRNA telomeric repeat-containing RNA (TERRA) can regulate telomere and mitochondrial function in the pathogenesis of IPF. They found that TERRA was highly expressed in peripheral blood mononuclear cells of IPF patients, and the TERRA expression levels were inversely correlated with FVC%. Telomerase can prolong telomeres and slow down cell senescence [41]. The progression of IPF is closely related to apoptosis, and mitochondria are the control center for apoptosis and oxidative stress. The study results have shown that TERRA inhibits telomerase activity and promotes the progression of IPF. RNA interferes with TERRA expression can regulate mitochondrial function and improves the function of oxidative stress-related genes (reactive oxygen species, superoxide dismutase, and catalase) and apoptosis-related genes (cytochrome-c, caspase-9, and caspase-3). Therefore, they believe that regulation of TERRA expression may become one of the IPF treatment pathways.

### 3.3 Interactions between lncRNAs and miRNAs and their regulatory networks in IPF

Currently, high-throughput sequencing technology was used to analyze the differentially expressed miRNAs in normal lung tissues and lung tissues of IPF patients, and then GO annotation and KEGG pathway enrichment analysis were used to screen the molecular targets most related to the diagnosis and prognosis of IPF, which laid the foundation for the follow-up clinical transformation therapy. We believe that identifying the diverging interaction network of lncRNAs and miRNAs orchestra and genes will provide important clues for understanding the mechanism of IPF and developing novel diagnostic and therapeutic strategies. At the same time, most of the latest progress of understanding of miRNA and lncRNA regulation in pulmonary fibrosis in the past 3 years are summarized and presented in Tables 1 and 2, respectively.

**Table 1:** Differential expression of miRNA in IPF

No.	Region	Sample	Target miRNA	Subjects (*n*)	Function	Ref.
1	Germany	Human lung tissues	Lethal 7d (MIRLET7D)	Control = 5	Treatment	[47]
				IPF = 10		
2	Belgium	Human lung tissues	ME1-16	Control = 108	Treatment	[48]
				IPF = 160		
3	China	BAL	microRNA‑30a	Control = 16	Treatment	[49]
				IPF = 30		
4	China	Human plasma	miR‑324‑5p miR‑630	Control = 10	Treatment	[17]
				IPF = 10		
5	Belgium	Human sputum	miR-142-3p	Control = 14	Diagnosis	[18]
				IPF = 16		
6	China	Human lung tissues	miR-409-5p has-miR-376c	Control = 25	Treatment	[50]
				IPF = 23	Diagnosis	
7	China	Human blood	miR-708-3p	Control = 78	Treatment	[15]
				IPF = 78		
8	Mexico	Fibrotic primary fibroblast cells	miRNA-21	Control = 6	Treatment	[51]
				IPF = 6	Diagnosis	
9	Greece	BAL	miR-185 miR-29a	IPF = 57	Treatment	[21]
				LC = 32		
10	China	Rat lung tissues	miR-541-5p	Rat	Treatment	[23]
11	California	Mouse lung tissues	miRNA-29c	Control = 4	Treatment	[14]
		Human lungs tissues		IPF = 7		
12	China	Human blood	miR-30a	Control = 46	Treatment	[52]
				IPF = 46		
13	Texas	Alveolar epithelial cells	miR-34a	Cells	Treatment	[53]
14	China	Rat pleural mesothelial cells	miR-18a-5p	Cells	Treatment	[24]
15	China	Lung resident mesenchymal stem cells	miR-497-5p	Cells	Treatment	[54]
16	Birmingham	Mice lung tissues	miR-34a	Mice	Treatment	[55,56]
17	USA	Bronchial epithelia	miR-323a-3p	Lung transplant patients = 11	Treatment	[22]
				Lung transplant patients with BOS = 7		
18	China	Plasma	miR-25-3p let-7d-5p	AE-IPF = 3	Diagnosis	[57]
				S-IPF = 3		
				Control = 3		
19	China	Mice lung tissues	miR-29b	Mice	Treatment	[58]
20	Japan	Human serum	miR-21-5p	Control = 21	Prognosis	[59]
				IPF = 41		
21	Italy	Human lung tissues	miR-200	Control = 34	Diagnosis	[60]
				IPF = 34		
22	China	Human lung tissues	miR‑221	Control = 10	Treatment	[61]
				IPF = 10		
23	Japan	Human lung tissues	miR-29a	Control = 17	Treatment	[62]
				LC with IPF = 8	Diagnosis	
24	China	Lung resident mesenchymal stem cells	miR-877-3p	Cells	Treatment	[20]
25	Indiana	Human lung tissues	miR-185 miR-186	Control = 15	Treatment	[63]
				IPF = 15		
26	China	Human lung tissues	miR-130b-3p	Control = 3	Treatment	[29]
				IPF = 4		
27	China	Human lung tissues	miR-26a	NM	Treatment	[64]
28	Birmingham	Human lung fibroblasts	miR-27a-3p	Cells	Treatment	[13]

LC: lung cancer; BOS: bronchiolitis obliterans syndrome; AE-IPF: acute exacerbation of idiopathic pulmonary fibrosis; S-IPF: stable of idiopathic pulmonary fibrosis; NM: not mentioned.

**Table 2:** Differential expression of lncRNA in IPF

No.	Region	Sample	Target lncRNA	Regulated miRNA/genes	Subjects (*n*)	Function	Ref.
1	China	Mice lung fibrosis tissues	H19	miR-196a/COL1A1	Mice	Treatment	[4]
2	China	Human peripheral blood	TERRA	Telomeres; mitochondria; associated genes; components associated with telomeres; mitochondria-associated cyclin E gene	Control = 24	Treatment	[40]
					IPF = 24		
3	China	Sprague Dawley (SD) rats	lnc-PCF	miR-344a-5p/map3k11	Rats	Treatment	[43]
4	China	The primary lung fibroblasts	PFAR	miR-15a/YAP1-Twist1	Cells	Treatment	[65]
5	China	Human lung tissues	H19	miR-140/Smad3	Control = 15	Treatment	[3]
					IPF = 15	Diagnosis	
6	China	Human lung tissues	ZEB1-AS1	miR-141-3p/ZEB1	NM	Treatment	[66]
7	China	Blood	lncITPF	ITGBL1	Control = 76	Treatment	[39]
					IPF = 76	Diagnosis	
8	China	Mice fibrotic hearts	PFAR	miR-138/YAP1	Mice	Treatment	[42,67]
9	China	Mouse lung fibroblasts	PFRL	miR-26a/Smad2	Cells	Treatment	[68]
10	China	Mice lung tissues	lncRNAPCAT29	miRNA-221/N4bp2; Plxna	Mice	Treatment	[69]
11	China	Mice lung tissues	lncRNA-ATB	miR-200c/ZEB1	Mice	Treatment	[70]
12	China	Blood	AP003419.16	RPS6KB2	Control = 4	Treatment	[38]
					IPF = 4		
13	China	Mice lung tissues	MALAT1	miR-503/PI3K p85	Mice	Treatment	[71]
14	China	Mouse fibroblast cells	H19	miR-29b/COL1A1; Acta2	Cells	Treatment	[58]
15	China	Mice lung tissues	CHRF	miR-489/MyD88; Smad3	Mice	Treatment	[72]
16	China	Mouse fibroblast cells	lincRNA-p21	Thy-1	Cells	Treatment	[73]
						Diagnosis	

NM: not mentioned.

It has been found that lncRNAs with target sequences similar to miRNAs can regulate the abundance of RNA by isolating miRNAs, or that lncRNAs can directly bind to miRNAs and regulate their functions. Although miRNAs and lncRNAs play an important role in the occurrence and progression of IPF, their regulatory networks in IPF remain unclear. Therefore, numerous studies [4,42,43] have been carried out on the intrinsic regulatory networks of lncRNAs and miRNAs in the pathogenesis of IPF.

A recent study [44] showed that lncRNA NONMMUT021928 (PFAL) competitively binds to miR-18a and promotes pulmonary fibrosis through CTGF gene. Li et al. [44] first found that PFAL was significantly upregulated in pulmonary fibrosis mice and TGF-β1-induced fibrotic pulmonary fibroblasts. The *in vitro* and *in vivo* experiments further demonstrated that overexpression of PFAL promoted the proliferation, migration, myofibroblastic transformation, extracellular matrix deposition, and fibrosis of lung fibroblasts by modulating miR-18a. On the contrary, the above fibrosis indexes were all decreased after the PFAL was knocked out. Further, they studied the intrinsic mechanism and showed that PFAL played the role of an endogenous RNA, competitively binds to miR-18a, and inhibits its expression and activity, thereby promoting lung fibroblast activation and fibrosis. In addition, they found that knock-out of miR-18a resulted in fibrosis of lung fibroblasts, whereas its overexpression reduced TGF-β1-induced pulmonary fibrosis. At the same time, the expression of miR-18a was decreased in patients with IPF by RT-PCR. Therefore, they believe that by knocking out PFAL and then regulating the expression of miR-18a, it may become a new treatment for pulmonary fibrosis.

Liu et al. [43] found that a novel lncRNA BC158825 (lnc-PCF) can target miR-344a-5p, thereby regulating its target gene map3k11 to promote pulmonary fibrosis. They first performed microarray analysis of fibrotic lung tissue and normal lung tissue, and the results showed that lnc-PCF was significantly expressed compared to other transcripts. Subsequently, the epithelial cells’ knocked-out and knocked-in lnc-PCF were detected by real-time cell analysis system, flow cytometry, and western blot analysis. The results showed that lnc-PCF promoted TGF-β1-induced epithelial cell proliferation. lncRNA can be targeted to regulate the function of miRNAs. Therefore, they based on TargetScan, miRanda data, and miRbase and used bioinformatics to predict the target miRNA of lnc-PCF. The predictive miRNAs were verified by qRT-PCR in fibrotic lung tissue and fibrotic cells, and ultimately determined that microRNAs-344a-5p were potential targets of lnc-PCF. miRNAs function by interacting with target genes. Consequently, they predicted the target gene of miR-344a-5p by the same method and determined that map3k11 as an important target gene of miR-344a-5p. To evaluate the therapeutic effect of interfering with lnc-PCF, adenovirus encapsulated with interfered sequence of lnc-PCF (sh-lnc-PCF) was injected into lung tissue of rats with pulmonary fibrosis. The results showed that the indexes of pulmonary fibrosis in sh-lnc-PCF group were significantly lower than those in control group, which confirmed that lnc-PCF was one of the potential targets for IPF treatment.

In addition, Lu et al. [4] took lncRNA H19 as a research target for the pathogenesis of IPF. They found that the expression of H19 was significantly increased in TGF-β-induced fibroblasts and BML-induced pulmonary fibrosis. Then, the target miRNAs of H19 were predicted by exploring Starbase v2.0 (http://starbase.sysu.edu.cn) and verified by dual-luciferase reporter assay. The results showed that miR-196a was a target of H19 in IPF. COL1A1 is a known target gene of miR-196a. To explore the relationship between H19, miR-196a, and COL1A1, they found that the expression of COL1A1 decreased with the decrease in H9, which could be reversed by microRNA-196a inhibitors. Therefore, they believe that H19 may competitively bind to microRNA-196a to regulate COL1A1 expression. Finally, *in vitro* and *in vivo* results have shown that downregulation of H19 attenuates lung fibroblast activation and pulmonary fibrosis, the phenomenon that can be reversed by miR-196a inhibitors. This confirmed once again that lncRNA H19 could promote the expression of COL1A1 through the competitive binding of microRNA-196a and accelerate the process of pulmonary fibrosis. Therefore, they believe that H19 plays an important role in the pathogenesis of IPF, and H19 is one of the directions of IPF gene-targeted therapy.

## 4 Conclusions

In most studies, total RNA was extracted from lung tissue or blood samples of IPF patients and normal people, and transcriptome sequencing (including microRNAs and lncRNAs sequencing) was performed [19,45]. The sequencing results were subjected to bioinformatics analysis and enrichment analysis to classify differentially expressed miRNAs or lncRNAs, and one or more important miRNAs (or lncRNAs) were selected as research objects. Further, target miRNAs (or genes) were identified by various experimental techniques such as exploring database, RNA fluorescence *in situ*, and ELISA. Finally, the *in vivo* or *in vitro* experiments were conducted to verify the role of target miRNAs, target lncRNAs, and target genes in the pathogenesis of IPF by qRT-PCR, western blot, and transfection. The research model is reasonable, but there are also certain limitations. One miRNA may regulate the expression of multiple genes, and the same gene may be regulated by multiple miRNAs. This phenomenon also applies to lncRNAs. Therefore, this research model can clearly explain one of the modes of action but failed to link the multiple modes of IPF.

Further need to demonstrate the correlation between gene expression and disease occurrence. In brief, the safety of gene therapy. For instance, Du et al. [46] found that the expression of lncRNA CDKN2B-AS1 and adjacent gene CDKN2A in peripheral blood of patients with IPF was significantly downregulated and activated p53 signaling pathway to promote the formation of lung cancer. Therefore, the follow-up study needs to further explore gene target therapy for IPF patients while avoiding the occurrence of cancer or fatal diseases.

In addition, there is currently no uniform method for miRNA or lncRNA isolation, and the specimens used to extract total RNA mainly come from lung tissue or blood specimens of IPF patients. The extraction methods of these specimens belong to invasive examinations. Moreover, research on biological samples such as saliva and sputum in IPF patients is still less; therefore, the detection of miRNA expression in these samples to diagnose IPF needs further investigation. At present, there is no treatment for IPF etiology or reversal of IPF. The role of ncRNAs in the pathogenesis of IPF requires a lot of research to explore, which provides a new scheme for molecular targeted therapy of IPF.
